# Histidine-rich glycoprotein prevents septic lethality through neutrophil regulation

**DOI:** 10.1186/cc14026

**Published:** 2014-12-03

**Authors:** M Nishibori, H Wake, S Mori, K Liu, Y Morioka, K Teshigawara, M Sakaguchi, K Kuroda, H Takahashi, A Ohtsuka, T Yoshino, H Morimatsu

**Affiliations:** 1 Department of Pharmacology, Okayama University Graduate School of Medicine, Dentistry, and Pharmaceutical Sciences, Okayama, Japan; 2 Department of Pharmacology, Faculty of Pharmaceutical Science, Shujitsu University, Okayama, Japan; 3 Department of Molecular Biology, Okayama University Graduate School of Medicine, Dentistry, and Pharmaceutical Sciences, Okayama, Japan; 4 Department of Anesthesiology, Okayama University Graduate School of Medicine, Dentistry, and Pharmaceutical Sciences, Okayama, Japan; 5 Department of Pharmacology, Faculty of Medicine, Kinki University, Osakasayama, Japan; 6 Department of Human Morphology, Okayama University Graduate School of Medicine, Dentistry, and Pharmaceutical Sciences, Okayama, Japan; 7 Department of Pathology, Okayama University Graduate School of Medicine, Dentistry, and Pharmaceutical Sciences, Okayama, Japan

## Introduction

Although there have been many clinical trials for treatment of sepsis, no drugs are available at present. Sepsis is thought to be complex disorders; activation/lesion of vascular endothelial cells, accelerated coagulation and platelet aggregation, leukocyte activation or paralysis, and hypercytokinemia. To treat the pathological status of sepsis, it must be necessary to control these plural disorders simultaneously or sequentially. In the present study, we identified and characterized a plasma protein histidine-rich glycoprotein (HRG) as a factor that decreases dramatically in septic condition and maintains neutrophil's shape and functional quiescence. We clarified the involvement of HRG in septic pathogenesis and propose a novel therapy for sepsis based on that.

## Methods

Sepsis was induced in mice by cecal ligation and puncture. The mice were treated with HRG purified from human plasma after confirming the marked decrease in plasma HRG. We evaluated the beneficial effects of HRG administration on survival rate, lung inflammation, and the state of circulating neutrophils using *in vivo *imaging. Purified neutrophils from human blood were treated with HRG and analyzed with respect to neutrophil shape, adhesiveness to vascular endothelial cells, passage through microcapillaries, production of reactive oxygen species, and cytoskeleton rearrangement with relevant signal transduction.

## Results

Supplementary treatment of septic mice with exogenous HRG for the decrease in plasma HRG improved the survival of mice remarkably. HRG treatment reduced the number of neutrophils in the lung, on which platelet aggregation and fibrin deposit were observed. HRG also inhibited the expression of mRNAs of IL-6, TNFα, iNOS, and PAI-1 in the lung. In contrast, knockdown of HRG by siRNA exacerbated lethality. Purified human HRG reversibly induced morphological changes in human neutrophils *in vitro*; induction of spherical shape with reduced microvilli and adhesiveness to vascular endothelial cells. HRG maintained the passage of neutrophils through microcapillaries and abolished the production of reactive oxygen species whereas HRG had no effect on the expression of adhesion molecules including CD11b and CD62L.

## Conclusion

We show that plasma protein HRG is a crucial factor that keeps the circulating neutrophils quiescent and prevents unnecessary activation in bloodstream using a cecal ligation and puncture model in mice and human neutrophils. The attachment of neutrophils on the vascular wall under reduced concentration of HRG was accompanied by microthrombus formation in the lung, leading to ARDS. Supplementary therapy with HRG may thus provide a novel strategy for the treatment of septic patients through neutrophil regulation.

